# Psychological Health Conditions and COVID-19-Related Stressors Among University Students: A Repeated Cross-Sectional Survey

**DOI:** 10.3389/fpsyg.2021.741332

**Published:** 2022-01-05

**Authors:** Maria Clelia Zurlo, Maria Francesca Cattaneo Della Volta, Federica Vallone

**Affiliations:** ^1^Dynamic Psychology Laboratory, Department of Political Science, University of Naples Federico II, Naples, Italy; ^2^Department of Humanities, University of Naples Federico II, Naples, Italy

**Keywords:** COVID-19 pandemic, psychological health, repeated cross-sectional survey, stress, university students

## Abstract

The Coronavirus Disease 2019 (COVID-19) pandemic has broadly impacted university students’ customary life, resulting in remarkable levels of stress and psychological suffering. Although the acute phase of the crisis has been overcome, it does not imply that perceived stress related to the risk of contagion and to the changes in the relational life experienced over more than 1 year of the pandemic will promptly and abruptly decrease. This study aims at comparing university students’ psychological health conditions before and during the COVID-19 pandemic, but also at providing information on how psychological health conditions evolved over the 1 year of the pandemic. We analyzed data from a repeated cross-sectional survey on different samples of university students before the pandemic in 2017 (*n* = 545) and during the pandemic (*n* = 671). During the pandemic, data were collected at three stages (Stage 1, April 2020 *n* = 197; Stage 2, November 2020 *n* = 274; and Stage 3, April 2021 *n* = 200). The COVID-19 Student Stress Questionnaire (CSSQ) and the Symptom-Checklist-90-Revised (SCL-90-R) were used to assess, respectively, COVID-19-related stressors (Relationships and Academic Life, Isolation, and Fear of Contagion) and the presence of psychological symptoms. Psychological health conditions were compared at baseline and during the pandemic, whereas both psychological health conditions and perceived levels of COVID-19-related stressors were compared over the three pandemic stages. In addition, Logistic Regression was used to explore the associations between COVID-19-related stressors and psychological symptoms. Findings revealed a significant increase in symptoms of Depression (DEP), Phobic-Anxiety (PHOB), Obsessive-Compulsive (O-C), and Psychoticism (PSY) from pre to during the pandemic. Perceived levels of COVID-19-related stress and specific psychological symptoms significantly increased as the pandemic was progressing. COVID-19-related stressors emerged as significantly associated with several psychopathological symptoms. Findings are discussed with the aim of providing tailored interventions to prevent mental disease and promote psychological adjustment in this specific stage of transition within this exceptional global emergency.

## Introduction

Over the last year, the spread of the Coronavirus Disease 2019 (COVID-19) and the consequent containment measures, which have been internationally adopted have significantly and extensively challenged people’ customary life, resulting in notable levels of psychological suffering reported by people worldwide (Cavalera, 2020; Lima et al., 2020; Rajkumar, 2020; Rossi et al., 2020; Becerra-García et al., 2021; Bueno-Notivol et al., 2021), and showing, in some cases, doubling and tripling of the prevalence of common mental illnesses (Pierce et al., 2020; Winkler et al., 2020). From this perspective, several studies have underlined the detrimental psychological impact of COVID-19 and containment measures, revealing high perceived loneliness, hopelessness, reduced life satisfaction, fatigue, and health anxiety not only among the health care workers, who are frontline facing the emergency, but also across the general population (Wallace et al., 2020; Duong, 2021; Mansueto et al., 2021).

In the last decade, growing research attention was given to university students’ mental health, and previous research carried out among this specific population across the world have revealed, even before the pandemic, increasing rates of psychological suffering (Zivin et al., 2009; Auerbach et al., 2018). Indeed, entering university represents a critical period for life development due to the different changes and challenges to be faced, i.e., the transition from adolescence to adulthood; the adjustment of the family roles/relationships; the achievement of independence; the restructuring of the social network by the inclusion of new relationships both in the academic (professors, university colleagues) and in the private life (friends, partner); the process of adapting to new academic/social demands (Sussman and Arnett, 2014; Saleh et al., 2017).

Over the last year, the spread of COVID-19 pandemic has imposed even more changes and challenges in university students’ daily life (Aristovnik et al., 2020). Specifically, several studies have broadly explored the impact of the COVID-19 on university students’ lives, underlining a wide spread of different psychological symptoms (Li et al., 2021), such as stress and difficulties in concentrating (Son et al., 2020; Zurlo et al., 2020; Baltà-Salvador et al., 2021; Lardone et al., 2021; Somma et al., 2021), anxiety and depression (Cao et al., 2020; Husky et al., 2020; Galvin et al., 2021; Rusch et al., 2021), eating disorders, alcohol/substance abuse (Gritsenko et al., 2020; Browning et al., 2021; Charles et al., 2021), sleep disorders (Debowska et al., 2020), and suicidal behaviors (Xu et al., 2021).

From this perspective, the containment measures undertaken worldwide – started from March 2020 and protracted for over 1 year – have deeply affected the academic context, by prescribing the closure of universities, the evacuations of campuses and accommodations, and the re-scheduling of the activities/events, with all the interactions (formal/informal) radically and exclusively transposed onto online platforms (United Nations Educational, Scientific and Cultural Organization, 2020). All these circumstances, therefore, implied wide and long-lasting modifications in students’ lives.

In particular, research conducted over the last year among university students has highlighted that the COVID-19 pandemic-related experiences induced fears due to the contagion risk (Rodríguez-Hidalgo et al., 2020; Yang et al., 2021), perceived stress related to the condition of social isolation (Filho et al., 2021), as well as to the extensive changes in their daily routine, mainly with respect to the relational domain. Indeed, the pandemic has implied wide modifications in key aspects of students’ customary lives, influencing (limiting/intensifying) all relationships both within the academic context (e.g., professors and colleagues) and within the private domain (e.g., friends, relatives, and partner; Zurlo et al., 2020). From this perspective, despite the massive rely on Information and Communication Technology (ICT) during the pandemic represented a key resource (allowing the fulfillment of the educational path and contacts with people outside their own home), this however entailed further potential risks for students’ psychological health (i.e., long hours on screens/working from home in shared and/or inappropriate spaces; unavailability of technological devices; lack of reliability of the internet connection; difficulties in using online platforms for distance learning; difficulties in “disconnecting” from the virtual world; Aguilera-Hermida, 2020; Islam et al., 2020; Kiraly et al., 2020; Papouli et al., 2020). Therefore, research also underlined the detrimental effects of perceived stress linked to the extended and exclusive use of technology, mainly in terms of increased anxiety and depression (Galvin et al., 2021).

Currently, despite the containment measures are gradually loosened worldwide, the COVID-19 and the associated restrictions are expected to have enduring consequences on mental health (Daly et al., 2021). This issue is particularly relevant also in light of research underlining the negative effects of students’ psychological suffering linked to the pandemic on several aspects of their academic life, in terms of difficulties in concentrating (Son et al., 2020), as well as reduction of self-efficacy, commitment/engagement, and academic performance (Aguilera-Hermida, 2020; Talsma et al., 2021).

Therefore, considering that previous studies exploring the effects of comparable periods of epidemics and quarantine [e.g., the Severe Acute Respiratory Syndrome (SARS) and the Middle East Respiratory Syndrome (MERS) outbreaks] underlined that, without timely psychological assessment and interventions, the prevalence of psychological disease may significantly escalate (Lam et al., 2009; Mak et al., 2009; Liu et al., 2012), there is an increasing need of developing longitudinal studies to assess and to monitor the perceived levels of stress and the psychological consequences of this unique crisis to target interventions (Becerra-García et al., 2021).

In this direction, longitudinal or repeated cross-sectional studies have been developed among university students from Poland (Debowska et al., 2020), Switzerland (Volken et al., 2021), and China (Zhang et al., 2020), revealing an increase in psychological disease, even if they only assessed psychological health during the early stages of the pandemic. Moreover, these studies lack a reliable baseline analysis against which it compares the prevalence of mental disorders to, and, to the best of our knowledge, research that compared pre to during pandemic mental health conditions refers to the general population, without specifically addressing the population of university students (Pierce et al., 2020; Winkler et al., 2020; Daly et al., 2021). Therefore, the present study has a two-fold objective. It aims at comparing university students’ psychological health conditions before and during the pandemic and at exploring how perceived levels of COVID-19-related stressors and psychological health conditions evolved over the 1 year of the pandemic (from April 2020 to April 2021). Moreover, considering the potential effects of enduring changes in all relationships within the private (e.g., relatives, friends, and partner) and academic domains (e.g., professors and colleagues), as well as of perceived isolation due to the lockdowns and fear of contagion, we also analyzed the associations between these specific COVID-19-related stressors and the development of psychopathological symptoms among university students.

In line with the study aims, the following research questions were proposed and tested:

*Research Question One* (*RQ1*): Are there differences in psychological health conditions reported by university students before (2017) and during (April 2020–2021) the pandemic?
*Research Question Two* (*RQ2*): Are there differences in perceived levels of COVID-19 related stressors and psychological health conditions reported by university students according to the three study stages (April 2020; November 2020; and April 2021) over the 1 year of the pandemic?
*Research Question Three* (*RQ3*): Are there significant associations between COVID-19-related stressors and psychological symptoms among university students?

This study could foster the development of more tailored psychological assessment, interventions, and follow-up taking into account university students’ pathways of adjustment to the pandemic emergency and capturing factors influencing the potential escalation of psychological suffering in the long term. Indeed, this may help to organize tailored support services and counseling interventions to promote students’ psychological health and, in turn, foster commitment and motivation, prevent withdrawal and dropout, and support a better adjustment to the academic and relational life.

## Materials and Methods

### Participants and Sampling

Over the period from April 2020 to April 2021, different samples of university students from Southern Italy were contacted three times, corresponding to the three main peaks of the pandemic (Stage 1, April 2020; Stage 2, November 2020; and Stage 3 April 2021). The three pandemic peaks varied in the number of COVID-19 confirmed cases/deaths as in the type of containment measures adopted [Istituto Superiore di Sanità (ISS), 2021; World Health Organization (WHO), 2021]. In particular, in April 2020, students were experiencing the first lockdown-type control measures, including the closing of universities, the cancelling of all events and the first semester of distance learning. In November 2020, after an attempt to loosen the restrictions in October, students were experiencing a significant strengthening of lockdown-type measures, with the enduring of distance learning. There was also an increase in the number of cases (involving youth to a greater extent, given the decrease in the average age of infected people). Finally, in April 2021, students were experiencing the effect of the 1 year of the pandemic, restriction, and distance learning, with still increasing rates of cases due to the widespread of variants of the virus.

Students were asked by the authors of the present study to participate in an online survey (Online Microsoft Teams forms) *via* institutional and informal channels (i.e., academic mailing lists; social media groups; and Microsoft Teams channel). They were given information about the study aims and they were assured about the confidentiality of the data. Research was performed in accordance with the 1964 Helsinki declaration and its later amendments or comparable ethical standards, and it was approved by the Ethical Committee from the University where the study took place (IRB: 12/2020). Overall 671 university students (Stage 1, April 2020 *n* = 197; Stage 2, November 2020 *n* = 274; and Stage 3 April 2021 *n* = 200) completed the questionnaires online. For the baseline analysis, data collected in 2017 with a self-selected sample of 545 university students from Southern Italy were used. The survey conducted in 2017 raised in the context of a wider research project – still carried out by the authors of the current study – which aimed at identifying risk and protective factors predicting psychological health of university students. All the students (contacted before the pandemic and during the pandemic) provided the informed consent. The two samples matched for Gender (Pre-Pandemic Women *n* = 409, 75.0%; During-Pandemic Women *n* = 506, 75.4%) and Age (Pre-Pandemic Age *M* = 21.6, *SD* = 2.72; During-Pandemic Age *M* = 21.3, *SD* = 3.27).

### Measures

In both the 2017 and 2020 surveys, the form included background information and the Symptom Checklist-90-Revised (SCL-90-R; Derogatis, 1994; Prunas et al., 2010). The COVID-19 Student Stress Questionnaire (CSSQ; Zurlo et al., 2020) was also administered in the three stages (2020–2021).

#### COVID-19 Student Stress Questionnaire

The CSSQ (Zurlo et al., 2020) was used to assess perceived stress related to the COVID-19 pandemic lockdown. It consists of seven items on a five-point Likert scale ranging from zero (Not at all stressful) to four (Extremely stressful) divided into three subscales: Relationships and Academic Life (four items, e.g., “How do you perceive the relationships with your university professors during this period of COVID-19 pandemic?”; Cut-off = 7.69), Isolation (two items, e.g., “How do you perceive the condition of social isolation imposed during this period of COVID-19 pandemic?”; Cut-off = 5.56), and Fear of Contagion (one item, i.e., “How do you perceive the risk of contagion during this period of COVID-19 pandemic?”; Cut-off = 2.73). The scale provides a Global Stress score (seven items, range = 0–28; Cut-off = 14.59; Cronbach’s *α* = 0.71). Cut-off scores and reliabilities were provided by the Italian validation study (Zurlo et al., 2020).

#### Psychological Health Conditions

The SCL-90-R (Derogatis, 1994; Italian version: Prunas et al., 2010) was administered to assess self-reported symptoms of psychological disease. The scale comprises 90 items on a five-point Likert scale ranging from zero (Not at all) to four (Extremely) and divided into nine subscales: Anxiety (ANX; 10 items, Cronbach’s *α* = 0.84; Cut-off male = 0.91, Cut-off female = 1.31), Depression (DEP; 13 items, Cronbach’s *α* = 0.87; Cut-off male = 1.08, Cut-off female = 1.62), Somatization (SOM; 12 items, Cronbach’s *α* = 0.83; Cut-off male = 1.09, Cut-off female = 1.67), Interpersonal Sensitivity (I-S; 9 items, Cronbach’s *α* = 0.83; Cut-off male = 1.01, Cut-off female = 1.34), Hostility (HOS; 6 items, Cronbach’s α = 0.80; Cut-off male = 1.18, Cut-off female = 1.34), Obsessive-Compulsive (O-C; 10 items, Cronbach’s *α* = 0.82; Cut-off male = 1.41, Cut-off female = 1.61), Phobic-Anxiety (PHOB; 7 items, Cronbach’s *α* = 0.68; Cut-off male = 0.44, Cut-off female = 0.72), Psychoticism (PSY; 10 items, Cronbach’s *α* = 0.77; Cut-off male = 0.71, Cut-off female = 0.81), and Paranoid Ideation (PAR; 6 items, Cronbach’s *α* = 0.76; Cut-off male = 1.00, Cut-off female = 1.67). Cut-off scores and reliabilities were provided by the Italian validation study (Prunas et al., 2010).

### Data Analysis

The statistical analyses were carried out using Statistical Package for Social Science (SPSS) version 21. Preliminarily, descriptive statistics were conducted. Skewness and Kurtosis were used to judge the normality of data, considering values between −2 and +2 as falling in the acceptable range (George and Mallery, 2019). Given that Skewness and Kurtosis values for all the variables fell within the range of −2 to +2, indicating that the data are fairly normally distributed, the following analyses were carried out. Firstly, in order to address *Research Question One* (*RQ1*) on differences in university students’ psychological health conditions before and during the pandemic, *t*-tests were carried out to compare mean scores of psychological symptoms reported by students during the pandemic with those reported by students at the baseline. In order to address the risk of false positive in *t*-tests (i.e., the probability of apparently significant results arising from repeated statistical tests), Benjamini-Hochberg False Discovery Rate (FDR) multiple testing corrections were used (Benjamini and Hochberg, 1995). Secondly, in order to address *Research Question Two* (*RQ2*) on differences in perceived levels of COVID-19 related stressors and psychological health conditions reported by university students according to the three study stages during the pandemic, ANOVA tests were used along with Bonferroni’s *post hoc* tests. Afterward, the study variables were dichotomized into low and high levels referring to the clinical cut-off points reported by the Italian validation studies of the CSSQ (Zurlo et al., 2020) and of the SCL-90-R (Prunas et al., 2010) (see Measure section), and frequencies and percentages of students reporting low and high (clinically relevant) levels of COVID-19-related stressors and psychological symptoms were calculated and compared by the three stages (Cross-tabulations and *χ*^2^ analyses). Finally, in order to address *Research Question Three* (*RQ3*) on the associations between COVID-19-related stressors and psychological symptoms, Logistic Regression Analyses were conducted.

## Results

### Changes in Psychological Health Conditions During the Pandemic From the Baseline

Table 1 shows mean scores for psychological health conditions, as measured by the SCL-90-R, reported by students at baseline (pre-pandemic) and during the pandemic. Responding to *RQ1*, findings from *t*-tests revealed that, during the pandemic, university students reported significantly higher levels of Depression (*p* < 0.001), Phobic Anxiety (*p* < 0.001), Obsessive-Compulsive (*p* < 0.01), and Psychoticism (*p* < 0.05). Scores regarding symptoms of Anxiety, Somatization, Interpersonal-Sensitivity, Hostility, and Paranoid Ideation reported by students during the pandemic did not differ significantly from the baseline (pre-pandemic).

**Table 1:** Descriptive statistics for psychological health conditions among university students before the pandemic (*n* = 545) and during the pandemic (*n* = 671).

	Before the pandemic 2017	During the pandemic 2020–2021	
	Mean ± SD	Mean ± SD	*B-H p*-value^a^
**Psychological symptoms**			
Anxiety (ANX)	1.12 ± 0.76	1.19 ± 0.77	0.193
Phobic-anxiety (PHOB)	0.37 ± 0.50	0.60 ± 0.63	**0.000** ^***^
Depression (DEP)	1.24 ± 0.80	1.48 ± 0.82	**0.000** ^***^
Somatization (SOM)	1.03 ± 0.72	1.03 ± 0.75	0.945
Obsessive-compulsive (O-C)	1.38 ± 0.77	1.54 ± 0.81	**0.003** ^**^
Psychoticism (PSY)	0.65 ± 0.61	0.75 ± 0.60	**0.015** ^*^
Interpersonal-sensitivity (I-S)	1.05 ± 0.78	1.14 ± 0.76	0.086
Hostility (HOS)	0.93 ± 0.73	1.00 ± 0.74	0.175
Paranoid-ideation (PAR)	1.13 ± 0.83	1.12 ± 0.77	0.921

a Differences were determined by Student *t*-tests; B-H: Benjamini-Hochberg corrections.

* *p* < 0.05;

** *p* < 0.01;

*** *p* < 0.001.

### Changes in Perceived COVID-19-Related Stressors and Psychological Health Conditions Across the Three Study Stages During the Pandemic

Table 2 shows means scores for perceived levels of COVID-19-related stressors and psychological health conditions across the study stages during the pandemic.

**Table 2:** Descriptive statistics including means (M) and Standard Deviations (SD) for perceived levels of Coronavirus Disease 2019 (COVID-19)-related stressors and psychological health conditions across the three study stages during the pandemic.

	During the pandemic						ANOVA *F*	Comparison (s)^a^
	April 2020 (S1) *n* = 197		November 2020 (S2) *n* = 274		April 2021 (S3) *n* = 200			
	*M*	*SD*	*M*	*SD*	*M*	*SD*		
**Perceived COVID-19-related stressors**								
Relationships and academic life	**4.99**	2.58	**6.62**	2.95	**6.61**	3.21	**11.84** ^***^	S1 < S2^***^, S3^***^
Isolation	**3.71**	2.00	**4.73**	1.87	**4.81**	2.03	**11.21** ^***^	S1 < S2^***^, S3^***^
Fear of contagion	**1.79**	1.22	**2.15**	1.16	**2.59**	1.13	**11.61** ^***^	S1 < S2^*^, S3^***^; S2 < S3^**^
Global stress	**10.49**	4.32	**13.51**	4.52	**14.01**	4.85	**18.78** ^***^	S1 < S2^***^, S3^***^
**Psychological symptoms**								
Anxiety (ANX)	**1.03**	0.70	**1.16**	0.75	**1.44**	0.82	**7.09** ^***^	S1 < S3^**^; S2 < S3^**^
Phobic anxiety (PHOB)	**0.51**	0.58	**0.59**	0.62	**0.72**	0.66	2.83	-
Depression (DEP)	**1.24**	0.71	**1.49**	0.83	**1.71**	0.83	**7.94** ^***^	S1 < S2^*^, S3^**^
Somatization (SOM)	**0.91**	0.69	**1.00**	0.72	**1.26**	0.84	**6.15** ^**^	S1 < S3^**^; S2 < S3^**^
Obsessive-compulsive (O-C)	**1.46**	0.74	**1.54**	0.84	**1.59**	0.81	0.61	-
Psychoticism (PSY)	**0.67**	0.44	**0.73**	0.63	**0.88**	0.62	**3.17** ^*^	S1 < S3^*^
Interpersonal-sensitivity (INT)	**0.96**	0.65	**1.15**	0.80	**1.31**	0.71	**5.18** ^**^	S1 < S3^**^
Hostility (HOS)	**0.92**	0.67	**1.00**	0.75	**1.11**	0.76	1.54	-
Paranoid ideation (PAR)	**0.99**	0.69	**1.09**	0.78	**1.33**	0.77	**5.04** ^**^	S1 < S3^**^; S2 < S3^*^

S1, Stage 1; S2, Stage 2; and S3, Stage 3.

a Bonferroni test.

* *p* < 0.05;

** *p* < 0.01;

*** *p* < 0.001.

Responding to *RQ2*, findings from ANOVA and Bonferroni’s *post hoc* tests revealed a significant increase in perceived COVID-19-related stress and psychological symptoms reported in Stage 3 from those reported in Stage 1. In particular, with respect to COVID-19-related stressors, it emerged that perceived stress related to changes in Relationship and Academic Life, Isolation, and perceived Global Stress score in Stage 2 and in Stage 3 increased significantly from Stage 1. Moreover, perceived stress related to Fear of Contagion constantly increased as the pandemic was progressing, so that scores in Stage 2 and 3 increased significantly from Stage 1, as well as scores in Stage 3 increased significantly from Stage 2.

With respect to psychological health conditions, data revealed that perceived levels of Anxiety, Somatization, and Paranoid Ideation in Stage 3 increased significantly from Stage 1 and from Stage 2. Furthermore, perceived levels of Depression in Stage 2 and in Stage 3 increased significantly from Stage 1, whereas perceived levels of Interpersonal-Sensitivity, and Psychoticism in Stage 3 increased significantly from Stage 1. Finally – despite higher – perceived levels of Phobic-Anxiety, Obsessive-Compulsive, and Hostility did not statistically differ across the three stages of the pandemic.

Furthermore, noteworthy and increasing number of students reporting clinically relevant levels of COVID-19-related stress and psychological symptoms were also found across the three stages of the pandemic (Table 3). In particular, referring to data from the last stage (April 2021), it emerged that about one half of the sampled students reported clinically relevant levels of COVID-19-related stress, mainly related to Fear of Contagion (59.0%), but also to perceived Isolation (39.0%) and Relationships and Academic Life (38.0%). Considering the psychopathological symptoms underlined by using the SCL-90-R, it emerged that in Stage 3 about/more than half of students reported clinically relevant levels of Depression (57.5%) and Psychoticism (55.0%), followed by Obsessive-Compulsive (50.0%), Anxiety (46.5%), and Interpersonal-Sensitivity (46.5%). Notable number of students reporting clinical levels of Phobic-Anxiety, Paranoid-Ideation, Somatization, and Hostility were also found.

**Table 3:** Number and percentages of university students reporting low and high (clinically relevant) levels of perceived COVID-19-related stressors and psychological symptoms by the three study stages during the pandemic.

	During the pandemic						*χ* ^2^ ^a^
	April 2020 *n* = 197		November 2020 *n* = 274		April 2021 *n* = 200		
	*N*	(%)	*N*	(%)	*N*	(%)	
**Perceived COVID-19-related stressors**							
Relationship and academic life							
Low	166	(84.3)	171	(62.4)	124	(62.0)	
High	31	(15.7)	103	(37.6)	76	(38.0)	**17.20** ^***^
Isolation							
Low	150	(76.1)	170	(62.0)	122	(61.0)	
High	47	47 (23.9)	104	(38.0)	78	(39.0)	**7.15** ^*^
Fear of contagion							
Low	136	(69.1)	163	(59.5)	82	(41.0)	
High	61	(30.9)	111	(40.5)	118	(59.0)	**16.90** ^***^
Global stress							
Low	158	(80.2)	162	(59.1)	110	(55.0)	
High	39	(19.8)	112	(40.9)	90	(45.0)	**17.03** ^***^
**Psychological symptoms**							
Anxiety (ANX)							
Low	130	(66.0)	162	(59.1)	107	(53.5)	
High	67	(34.0)	112	(40.9)	93	(46.5)	3.12
Phobic anxiety (PHOB)							
Low	147	(74.6)	183	(66.8)	117	(58.5)	
High	50	(25.4)	91	(33.2)	83	(41.5)	5.21
Depression (DEP)							
Low	122	(61.9)	145	(52.9)	85	(42.5)	
High	75	(38.1)	129	(47.1)	115	(57.5)	**6.98** ^*^
Somatization (SOM)							
Low	164	(83.0)	210	(76.6)	135	(67.5)	
High	33	(17.0)	64	(23.4)	65	(32.5)	**6.32** ^*^
Obsessive-compulsive (O-C)							
Low	122	(61.9)	153	(45.8)	100	(50.0)	
High	75	(38.1)	121	(44.2)	100	(50.0)	2.58
Psychoticism (PSY)							
Low	128	(65.0)	173	(63.9)	90	(45.0)	
High	69	(35.0)	99	(36.1)	110	(55.0)	**11.97** ^**^
Interpersonal-sensitivity (INT)							
Low	147	(74.6)	175	(63.9)	107	(53.5)	
High	50	(25.4)	99	(36.1)	93	(46.5)	**9.07** ^*^
Hostility (HOS)							
Low	151	(76.6)	205	(74.8)	146	(73.0)	
High	46	(23.4)	69	(25.2)	54	(27.0)	0.351
Paranoid ideation (PAR)							
Low	149	(75.6)	194	(70.8)	126	(63.0)	
High	48	(24.4)	80	(29.2)	74	(37.0)	3.58

a Cross-tabulations and Chi-Square Analyses.

* *p* < 0.05;

** *p* < 0.01;

*** *p* < 0.001.

### Associations Between COVID-19-Related Stressors and Psychological Symptoms

Table 4 shows the associations between perceived COVID-19-related stressors and psychological symptoms among university students.

**Table 4:** Logistic Regression Analyses: Associations between COVID-19-related stressors and psychological symptoms among university students (*n* = 671).

Predictors	Relationships and academic life		Isolation		Fear of contagion	
	OR	CI	OR	CI	OR	CI
**Outcomes**						
Anxiety (ANX)	**3.2** ^***^	2.1–4.9	1.4	0.9–2.2	**1.5** ^*^	1.0–2.3
Phobic anxiety (PHOB)	**2.1** ^**^	1.3–3.2	1.2	0.8–1.8	**2.0** ^***^	1.8–4.1
Depression (DEP)	**4.2** ^***^	2.7–6.6	**1.6** ^*^	1.1–2.5	0.9	0.6–1.3
Somatization (SOM)	**3.4** ^***^	2.1–0.5.4	**1.7** ^*^	1.1–2.8	1.3	0.8–2.1
Obsessive-compulsive (O-C)	**2.8** ^***^	1.8–4.3	**1.7** ^*^	1.1–2.6	1.2	0.8–1.8
Psychoticism (PSY)	**2.6** ^***^	1.7–3.9	**1.7** ^**^	1.1–2.6	1.0	0.7–1.5
Interpersonal-sensitivity (INT)	**2.4** ^***^	1.5–3.6	**1.7** ^*^	1.1–2.5	1.0	0.7–1.6
Hostility (HOS)	**2.2** ^**^	1.4–3.6	**2.6** ^***^	1.6–4.1	0.6	0.4–1.0
Paranoid ideation (PAR)	1.4	0.9–2.2	1.3	0.8–2.0	0.7	0.5–1.1

OR, odds ratios; CI, confidence intervals.

* *p* < 0.05;

** *p* < 0.01;

*** *p* < 0.001.

Responding to *RQ3*, data revealed that high perceived levels of stress related to Relationships and Academic Life and to Fear of Contagion were significantly associated with high risk for reporting clinical levels of Anxiety and Phobic-Anxiety, while high perceived levels of stress related to Relationships and Academic Life and to Isolation were significantly associated with high risk for reporting clinical levels of Somatization, Obsessive-Compulsive, Depression, Hostility, Psychoticism, and Interpersonal-Sensitivity. No significant associations between perceived COVID-19-related stressors and Paranoid Ideation were found.

## Discussion

The present study aims at providing a greater understanding of the psychological impact of COVID-19 and containment measures among university students by comparing their psychological health conditions before and during the pandemic and by exploring how perceived levels of COVID-19-related stressors and psychological symptoms evolved over the 1 year of the global crisis (from April 2020 to April 2021). This, indeed, can support the development of timely and tailored interventions preventing the escalation of psychological disease in the context of this specific and delicate stage of transition within the COVID-19 global emergency.

Firstly, responding to *Research Question One* (i.e., exploring differences in students’ psychological health conditions before and during the pandemic), we found that symptoms of Depression, Phobic-Anxiety, Obsessive-Compulsive, and Psychoticism reported by students during the pandemic significantly increased from those reported in 2017, while perceived levels of Anxiety, Somatization, Interpersonal-Sensitivity, Hostility, and Paranoid Ideation remained similar. These findings were consistent with research examining mental health among the general population before and during the pandemic (Winkler et al., 2020; Becerra-García et al., 2021; Daly et al., 2021) and supported – also among the student population – the detrimental psychological impact of the COVID-19 outbreak. Nonetheless, these data also provided evidence on specific outcomes to be carefully considered in the context of the contemporary research and interventions related to the COVID-19, so helping to identify symptoms of individual and relational disease that may have been occurred in response to this new reality.

In this direction, responding to *Research Question Two* (i.e., exploring differences in students’ perceived levels of COVID-19 related stressors and psychological health conditions according to the three study stages during the pandemic), overall data confirmed the substantial exacerbation of psychological suffering over the 1 year of the outbreak, providing evidence matching concerns about the detrimental trend of mental disease escalation as the pandemic was progressing (Debowska et al., 2020; Zhang et al., 2020; Li et al., 2021; Volken et al., 2021).

Specifically, considering COVID-19-related stressors, our findings revealed that perceived stress related to Relationship and Academic Life and Isolation significantly increased from the beginning of the pandemic, with the main exacerbation peak from April 2020 to November 2020 (Stage 1–Stage 2), and a substantial invariance of the higher levels of stress reported in November 2020 and April 2021 (Stage 2–Stage 3). These results could potentially reflect the adjustment processes adopted by students to the pandemic condition; indeed, after an initial sharp increase in perceived stress linked to the drastic and abrupt changes in relationships and academic life and to the condition of isolation, we hypothesized that the enduring of this exceptional crisis may have resulted in attempts to adjust to this new restricted and modified life conditions. Nonetheless, such efforts may not necessarily imply a successful adaptation, and they may instead result in chronicization of stress and increased levels of several outcomes, as revealed by our data on students’ psychological health conditions.

From this perspective, these data can be also interpreted considering the specificities of the three major peaks of the pandemic, corresponding to the three stages of the present study. Italy was, indeed, the first European country to implement a national quarantine (full lockdown from March to June 2020; World Health Organization (WHO), 2021). It mandated isolation at home (“stay-at-home-order”) with permitted mobility for essential services or seeking medical care only. Schools and universities were all provisionally closed, resulting in growing stress and feelings of uncertainty among students (Lee 2020), which were experiencing the first semester of distance learning. Our data on the perceived stress that emerged in April 2020 reflect this stage of pandemic.

After summer, however, restrictions were gradually eased, to be abruptly worsened in October–November 2020 (e.g., activities prohibited except for work/health/urgent reasons; social-distancing), with higher education still adopting distance learning; the latter entailing both further risks (i.e., techno-stress) and resources (i.e., techno-sociality) due to the possibility to maintain – despite virtually – key relational contacts (Galvin et al., 2021). Our findings on the perceived stress that emerged in November 2020 reflect this stage of pandemic. From November 2020 to April 2021, in Italy, the majority of regions were still facing the enduring of lockdowns and containment measures, which kept challenging students’ academic life and the whole social and relational sphere, so determining a stabilization of these circumstances and the potential chronicization of perceived stress. Therefore, there is a clear need to deal with the psychological effects of more than 1 year of distance learning, isolation, restrictions, and emergency.

Still considering COVID-19-related stressors, our data revealed that stress related to Fear of Contagion significantly and constantly increased across the three stages. These data may be interpreted considering the progression of the pandemic, characterized worldwide by increasing number of diagnosed cases, hospitalizations, and deaths, together with the decrease in the average age of infected people [Istituto Superiore di Sanità (ISS), 2021; World Health Organization (WHO), 2021]. Moreover, with particular reference to the last period examined (April 2021), the widespread of variants of the virus and the several issues and delays in the vaccination campaign may have resulted in an even growing fear and uncertainty related to the contagion risk.

Overall, these data can explain the remarkable and growing number of students reporting clinically relevant levels of all COVID-19-related stressors and of psychological symptoms highlighted in the present study. In particular, when analyzing clinically relevant levels of psychological disease reported by students in April 2021, it emerged that nearly 60% of students reported clinical levels of Depression, probably reflecting the detrimental effects of stress experienced over the 1 year of the pandemic and containment measures. Indeed, these data were noteworthy higher than those reported in the Italian general population during the first wave of the pandemic (17.3%; Rossi et al., 2020), so supporting evidence on the growing COVID-19-related psychopathological risk in younger people to be timely addressed to reduce the risk of mental illness escalation (Peng et al., 2020).

In this perspective, still considering the last peak of the pandemic, our findings revealed, in line with previous studies, alarming clinical levels of Obsessive-Compulsive (Ji et al., 2020), Anxiety and Phobic-Anxiety (Faisal et al., 2021), as well as Interpersonal Sensitivity (Jiang, 2020), which could be largely linked to the measures taken for infection prevention and to the radical changes in students’ individual and relational life (avoiding face-to-face-contacts and crowned places; isolation and social distancing; hand-washing/checking). Furthermore, our findings also underlined that more than one half of the students reported clinically relevant levels of Psychoticism. However, despite previous evidence suggested the presence of reactive psychosis in the context of the pandemic, mainly as a consequence of COVID-19 diagnosis (Smith et al., 2020), our data should be interpreted with caution, given that the Psychoticism subscale from the SCL-90-R reflects a *continuum* from the psychotic disorder to symptoms of interpersonal alienation, the latter characterized by isolation and withdrawal from social life, which were largely prescribed and forcibly experienced over more than 1 year of isolation, restrictions, and adjustment to a new reality. In this perspective, data induced to reflect upon the pathologizing feature of the containment measures, which, in themselves, endorsed social isolation, alienation, and withdrawal.

From this perspective, responding to *Research Question Three* (i.e., exploring associations between COVID-19-related stressors and psychological symptoms), findings from Logistic Regression analyses highlighted that COVID-19-related stressors were significantly associated to the risk for reporting psychopathological symptoms among students. Specifically, data revealed that Relationship and Academic Life, which, as we have previously underlined, has become a chronic source of stress over the 1 year of the outbreak, emerged as associated with almost all SCL-90-R subscales, with particular reference to Depression, Anxiety, and Somatization. These data underlined how the COVID-19-related restrictions drastically impaired students’ relational domain (i.e., impossibility to benefit from living the university life; online-only relationships with professors and colleagues; new circumstances of co-habiting with relatives with nearly exclusive sharing time/spaces), potentially resulting in depressive and anxious symptoms and in somatic manifestations of suffering linked to feelings of losses and lack of control.

Similarly, our data suggested that students who perceived high levels of stress related to Isolation (i.e., enduring and chronic stress related to social isolation and to changes in intimacy/sexual life) were at higher risk of reporting clinical levels of mental health outcomes, particularly in terms of Hostility. This may be due to the negative effects of the prolonged lack of contacts and closeness with loved ones (non-cohabiting relatives/friends/partner), which resulted in the escalation of anger and frustration related to the COVID-19 experiences. These data should be carefully considered when defining interventions fostering students’ well-being, given that younger people and individuals who had a higher initial stress response to the pandemic were at increasing risk for hostility escalation (Duan et al., 2020).

Finally, students who perceived high stress related to both Relationship and Academic Life and Fear of Contagion were at higher risk of suffering from Anxiety and Phobic-Anxiety. This clearly highlighted how not only the stressors related to the fear of the virus but also the perceived relational stress related to the strict measures imposed to limit the spread of contagion may have led to increasing feelings of uncertainty, tension, and worry and to behavioral responses of avoidance in the attempt to control the risk of infection and to adjust to this exceptional condition.

In summary, within this complex portrait, we considered the findings from the present study could help to effectively foster the development of tailored interventions to prevent mental disease escalation as well as to counteract the adverse effects of COVID-19 on psychological health among university students. Indeed, by comparing university students’ psychological health conditions before and during the pandemic, by exploring how their mental health evolved over the 1 year of the crisis and by testing the associations between specific COVID-19-related stressors and psychopathological symptoms, findings highlighted those areas to be carefully considered within health promotion campaigns as well as within psychological settings during this delicate transition stage of the pandemic, which could potentially represent a key turning point toward a gradual recovery or, conversely, a further worsening of the emergency.

From this perspective, interventions should carefully take into account the consequences of lockdowns – protracted for more than 1 year – and should mainly target students particularly affected by the COVID-19 and its related restrictions and those lacking social networks, who are, indeed, at higher psychopathological risk. These include the offering of tailored initiatives and counseling services for students, on the one side, fostering the re-appraisals of the COVID-19-related experiences and the adjustment to this new reality and, on the one other side, preventing that future potential lockdowns further exacerbate the feeling of loneliness and the psychological burden (Mansueto et al., 2021; Voltmer et al., 2021). This can be achieved, for example, by preserving social contacts both face-to-face (i.e., professors, tutors, and counselor could maintain safe personal meetings at the universities) and online (i.e., the universities could provide support to improve students’ digital skills for effectively participating in distance learning), so reducing the negative impact of changes in relationships and academic life and of perceived isolation, fostering a sense of support and belonging to a community, and eventually reducing the risk of students’ drop-out.

Notwithstanding the strengths of this study, some limitations need to be addressed. Firstly, this study adopted a repeated cross-sectional design, so limiting the possibility to propose cause-effect relationships, as well as to achieve information on trajectories of students’ psychological health conditions at the individual level. Moreover, only students from southern Italy were assessed, reducing the generalizability of the results. Therefore, future studies using longitudinal data and based on larger and nationally representative samples are needed to support our findings. Secondly, the study explored COVID-19-related stressors and psychological symptoms only, without addressing some confounding variables, as preset or past psychotherapy, as well as other dimensions which may play as key resources or further hindrance for students’ psychological health. Accordingly, in order to further deepen COVID-19-related stress and well-being processes among students, future research could address the exploration of negative social emotions such as guilt and shame (Cavalera, 2020), which can be triggered in university students within the current pandemic period, and could also address potential confounding variables (e.g., preset or past psychotherapy). Moreover, future studies could investigate the role of individual characteristics (e.g., personality traits and coping strategies), as well as of further situational characteristics (e.g., both risk and protective factors related to the use of technology), and could also consider other risks such as suicidal behaviors (Xu et al., 2021) and substance and/or alcohol use disorders (Gritsenko et al., 2020). In the same perspective, this study addressed psychological symptoms as outcome variables, while the COVID-19 pandemic as well as the containment measures may have had a significant impact not only on students’ psychological health conditions but also on their academic path. Future research could therefore include academic performance, academic motivation, and leaving intention as outcome variables. Future studies could also consider comparing psychological health conditions reported by university students with those reported by other specific population groups (e.g., emerging adults not enrolled in university degree courses; employees) and also students from other countries.

In conclusion, the study provided evidence which may assist policymakers and healthcare professionals in effectively organize and develop tailored assessment and interventions fostering students’ adjustment during this transition stage of pandemic and preventing this unique emergency become overwhelming for university students’ mental health.

## Data Availability Statement

The raw data supporting the conclusions of this article will be made available by the authors, without undue reservation.

## Ethics Statement

The studies involving human participants were reviewed and approved by Ethics Committee of Psychological Research of the University of Naples Federico II. The patients/participants provided their written informed consent to participate in this study.

## Author Contributions

MCZ: study conception and design, interpretation of data, drafting of manuscript, and critical revision. MFCDV: analysis and interpretation of data and drafting of manuscript. FV: acquisition of data, analysis and interpretation of data, and drafting of manuscript. All authors contributed to the article and approved the submitted version.

## Funding

This work was supported by Erasmus+ Project Code: 2020-1-UK01-KA226-HE-094622.

## Conflict of Interest

The authors declare that the research was conducted in the absence of any commercial or financial relationships that could be construed as a potential conflict of interest.

## Publisher’s Note

All claims expressed in this article are solely those of the authors and do not necessarily represent those of their affiliated organizations, or those of the publisher, the editors and the reviewers. Any product that may be evaluated in this article, or claim that may be made by its manufacturer, is not guaranteed or endorsed by the publisher.
